# Serum zinc reference intervals and its relationship with dietary, functional, and biochemical indicators in 6- to 9-year-old healthy children

**DOI:** 10.3402/fnr.v60.30157

**Published:** 2016-04-05

**Authors:** Camila Xavier Alves, Naira Josele Neves de Brito, Karina Marques Vermeulen, Márcia Marília Gomes Dantas Lopes, Mardone Cavalcante França, Selma Sousa Bruno, Maria das Graças Almeida, José Brandão-Neto

**Affiliations:** 1 Department of Internal Medicine, Postgraduate Program in Health Sciences, Federal University of Rio Grande do Norte, Natal, RN, Brazil; 2 Department of Statistics, Federal University of Rio Grande do Norte, Natal, RN, Brazil; 3 Department of Physiotherapy, Federal University of Rio Grande do Norte, Natal, RN, Brazil; 4 Department of Clinical and Toxicological Analyses, Federal University of Rio Grande do Norte, Natal, RN, Brazil; 5 Department of Internal Medicine, Federal University of Rio Grande do Norte, Natal, Brazil

**Keywords:** zinc status, reference interval, biochemical indicators, dietary indicators, functional indicators, young children

## Abstract

**Background:**

Zinc is an important cause of morbidity, particularly among young children. The dietary, functional, and biochemical indicators should be used to assess zinc status and to indicate the need for zinc interventions.

**Objective:**

The purpose of this study was to determine the zinc status and reference intervals for serum zinc concentration considering dietary, functional, and biochemical indicators in apparently healthy children in the Northeast Region of Brazil.

**Design:**

The cross-sectional study included 131 healthy children: 72 girls and 59 boys, aged between 6 and 9 years. Anthropometric assessment was made by body mass index (BMI) and age; dietary assessment by prospective 3-day food register, and an evaluation of total proteins was performed. Zinc in the serum samples was analyzed in triplicate in the same assay flame, using atomic absorption spectrophotometry.

**Results:**

With respect to dietary assessment, only the intake of fiber and calcium was below the recommendations by age and gender. All subjects were eutrophic according to BMI and age classification. Zinc intake correlated with energy (*p=*0.0019), protein (*p=*0.0054), fat (*p<*0.0001), carbohydrate (*p=*0.0305), fiber (*p=*0.0465), calcium (*p=*0.0006), and iron (*p=*0.0003) intakes. Serum zinc correlated with protein intake (*p=*0.0145) and serum albumin (*p=*0.0141), globulin (*p*=0.0041), and albumin/globulin ratio (*p*=0.0043). Biochemical parameters were all within the normal reference range. Reference intervals for basal serum zinc concentration were 0.70–1.14 µg/mL in boys, 0.73–1.17 µg/mL in girls, and 0.72–1.15 µg/mL in the total population.

**Conclusions:**

This study presents pediatric reference intervals for serum zinc concentration, considering dietary, functional, and biochemical indicators, which are useful to establish the zinc status in specific groups. In this regard, there are few studies in the literature conducted under these conditions, which make it an innovative methodology.

Zinc is an essential micronutrient with many enzymatic functions. Zinc controls the synthesis of DNA, RNA, and proteins, as well as the metabolism of proteins, carbohydrates, and lipids. Gene expression, immune competence, cognitive functions, psychomotor development, growth, and physical development are also associated with the actions of zinc. Therefore, inadequate zinc intake has profound health consequences in all cycles of human life from conception to old age (1, 2).

The World Health Organization (WHO), the International Atomic Energy Association (IAEA), and the United Nations Children's Fund (UNICEF) reviewed and recommended available indicators of population zinc status for international use. Three types of indicators were considered: dietary, functional, and biochemical. These indicators can be used together in population and subgroup assessments to estimate the zinc status (3).

## Dietary indicators

Assessments of dietary zinc intake provide information on the dietary patterns of a population or subpopulation, which is associated with the adequacy or inadequacy of zinc intake (3). Serum zinc concentrations in a population also reflect the habitual intake of this micronutrient in the diet (4). The best indicators for population assessment are the prevalence or probability of zinc intake below the Estimated Average Requirement (EAR) from 24-h recall surveys, which are the most widely used dietary assessment methods (5). Therefore, low serum zinc concentrations can be used to indicate risk of zinc deficiency in a population when the prevalence or probability of inadequate intake is greater than 25% (3, 6) to 73% (7, 8), depending on the geographic region.

## Functional indicators

Height-for-age is an important indicator that can help establish the nutritional status of zinc deficiency (9). Although this indicator is not specific to zinc status in a certain way, it can help establish whether children within a population or subgroup are healthy or not using non-invasive methods (9). The prevalence of children with low-length- or height-for-age should be at least 20% of the age-specific median (−2 SD) of the reference population (10). Other parameters to analyze nutritional status include anthropometric measurements of weight-for-age and body mass index (BMI)-for-age using the growth curves published by the WHO (10). Functional indicators can be used in combination with biochemical and dietary assessment as indicators of zinc status. It is ideal for more than one type of indicator to be used together to obtain a best estimate of the risk of zinc deficiency in a population or in subgroups.

## Biochemical indicators

Serum zinc is currently the most widely used and accepted biomarker of the risk of zinc deficiency in the population despite the poor sensitivity and imperfect specificity of serum zinc testing (11) and is used to identify groups for whom interventions should be indicated (6, 11, 12). This biomarker is sensitive to supplementation and depletion of zinc (12, 13).

The National Health and Nutrition Examination Survey II (NHANES II) suggested that for male and female children between 3 and 9 years of age, the mean serum zinc concentration is 0.83±0.03 µg/mL and the cutoff for zinc deficiency is 0.65 µg/mL, for samples collected in the morning hours in a non-fasted state. Furthermore, these reference intervals differ among populations and should be established regionally and locally (14–16).

There are many confounds as age, gender, tourniquet, position of subject, time of day of blood collection, non-fasting status, diet, and others, which can interfere with the measurement of serum zinc (4, 11, 12). It is because of these potential sources of interference that the results of serum zinc concentration assessments are not necessarily identical, causing inter-laboratory differences (12).

Furthermore, zinc is transported in the serum bound principally to albumin (70%) and tightly to α-2-macroglobulin (18%). A small amount is bound to transferrin and ceruloplasmin, and complexed with amino acids, especially histidine and cysteine. Therefore, concurrent protein deficiencies may affect serum zinc concentrations (6).

The aim of this study was to establish the status of zinc, especially the reference interval, and its relationship with dietary, functional, and biochemical indicators in a population of healthy children in the Northeast Region of Brazil for whom there are limited data. Furthermore, the methodology as suggested by Benoist et al. (3) has not been used until now.

## Methods

### Subjects

Two hundred and twenty prepubescent children of both genders, aged 6–9, were recruited from four municipal schools located on the East and West of Natal City, Rio Grande do Norte, Brazil. This study was cross-sectional, and a non-probability convenience sample was used. The students were authorized by their parents or guardians to take part in the study, and the study was approved by the Onofre Lopes University Hospital Research Ethics Committee at the Federal University of Rio Grande do Norte (UFRN), Brazil (number 323/09). Moreover, the Universal Trial Number (UTN) is U1111-1169-7345.

### Selection criteria

Each child was examined at the Laboratory of Multidisciplinary Chronic Degenerative Diseases at UFRN by the same endocrinologist. All children were in the Tanner stage 1 for genital, breast, and pubic hair growth (17), with body weight, height, and BMI within the normal reference range for their age (10). Exclusion criteria included underweight; overweight; obesity; Tanner stage 2 or more; acute, chronic, infectious or inflammatory diseases; nutritional disorder; history of surgery; and use of vitamin or mineral supplements.

### Study group

**Fig. 1:**
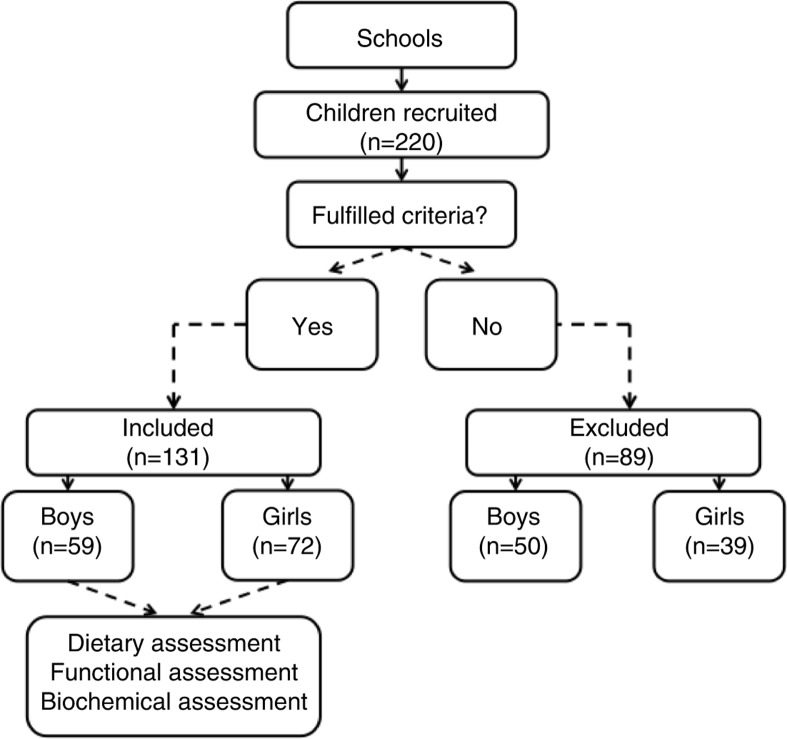
Design of the study group.

The volunteers were studied between 2009 and 2012. The study was completed with 59 male children and 72 female children (Fig. 1).

### Dietary indicators

Dietary assessment was performed with 92 children using a prospective 3-day food register on 2 weekdays and 1 day over the weekend. The parents were instructed to record all food and beverages consumed by the child. Calculations of energy, macronutrients, fiber, calcium, iron, and zinc were performed using the NutWin software version 1.5 (Nutrition Support Program) developed by the Department of Health Informatics, Federal University of São Paulo (UNIFESP), Brazil (18). Foods not included in the program were inserted based on food chemical composition tables (19). Two nutritionists performed these nutritional assessments.

### Functional indicators

Body weight (kg) and height (cm) were measured using an electronic balance (Balmak, BK50F, São Paulo, Brazil) and a stadiometer (Sanny Stadiometer Professional, American Medical of Brazil, São Paulo, Brazil), respectively.

The assessment of nutritional status was based on anthropometric indicators (weight-for-age, height-for-age, and BMI-for-age). This analysis was also based on BMI-for-age using the growth curves published by the WHO (10). We calculated this parameter accessing WHO AnthroPlus version 1.0.4 (20). Nutritionists performed anthropometric assessments, according to Dantas et al. (21).

### Biochemical indicators

#### Zinc

Zinc serum samples were analyzed in triplicate in the same assay by atomic absorption spectrophotometry (SpectrAA-240FS, Varian, Victoria, Australia) with a zinc cathode lamp according to the manufacturer's instructions. The sensitivity of zinc was 0.01 µg/mL, the coefficient of variation was 2.09%, and the reference range was 0.7–1.2 µg/mL, according to our laboratory assessment. The serum zinc concentration was determined from accuracy of the triplicate analyses and an internal reference standard.

Four milliliters of blood were collected for analysis of serum zinc. Venipuncture, collection, separation, and storage of zinc, including materials, chemicals, and laboratory procedures, were made according to Lopes et al. (22). The medical examination found no signs or symptoms of zinc deficiency.

### Total proteins and fractions

Total proteins, albumin, globulin, and albumin/globulin ratio were measured in 53 children by the colorimetric method in a biochemical analyzer with specific kits (Dade Behring Dimension AR, Deerfield, IL, USA).

### Statistical analyses

Data normality was assessed by the Kolmogorov–Smirnov test and normal Q–Q plot. Statistical analyses were performed using the Student's *t*-test for independent samples to compare continuous variables between boys and girls. Spearman's correlation was used between the variables, and the univariate linear regression model was used to test the association of serum zinc with serum proteins and fractions. The multivariate stepwise regression model was used to test the possibility of association between these proteins with zinc prediction variance. For the determination of serum zinc reference interval, the guidelines established by the International Federation of Clinical Chemistry (IFCC) were followed. These guidelines recommend that ≥120 individuals are used to estimate percentiles by the parametric method (23). The reference values were determined based on the 95% confidence interval, the lower value corresponding to the 5th percentile and higher value to the 95th percentile. Values are also reported as mean±SD, and *p*<0.05 was considered significant. Statistical tests were performed using the GraphPad Prism 6.0 software (San Diego, CA, USA), and Statistica 10.0 (StatSoft, Inc., Tulsa, OK, USA).

## Results

### Subjects

A total of 220 prepubescent children of both genders, aged 6–9, completed the study. The sample size of 131 children was adequate for the conclusions obtained in this study.

### Dietary indicators

Energy, protein, fat, carbohydrates, iron, and zinc intakes were adequate according to the Dietary References Intakes by age and gender of the children. However, the concentrations of fiber and calcium were below these recommendations (Table 1). There was no significant difference in zinc intake between boys (5.99±0.97 mg/day) and girls (5.99±1.11 mg/day). In addition, the mean zinc intake in the total population was 6.00±1.01 mg/day, which is within the normal reference range for the age group of 6–9 years.

**Table 1:** Energy and nutrient intake compared with its recommendations by age and sex

			95% CI		
				
Variable	Intake value	Mean diff	Lower	Upper	Reference value
Energy (kcal/day)	1,416±305.60	359.50	91.00	−90.00	6–9 years (boys): 1,573–1,978 kcal/day (24)
	1,571±233.70	204.50	70.00	−70.00	6–9 years (girls): 1,428–1,854 kcal/day (24)
Protein (g/kg/day)	1.7±0.48	0.95	0.10	−0.11	4–8 years (both sexes): 0.76 g/kg/day (25)
					9–13 years (both sexes): 0.76 g/kg/day (25)
Fat (g/day)	37.14±7.890	ND	ND	ND	ND (25)
Carbohydrate (g/day)	183.6±30.22	83.60	6.20	−6.30	100 g/day (25)
Fiber (g/day)	10.7±1.95	−14.32	0.52	−0.52	4–8 years (both sexes): 25 g/day (25)
	10.6±1.18	−15.36	0.63	−0.63	9–13 years (girls): 26 g/day (25)
	11.1±2.05	−19.89	1.02	−1.02	9–13 years (boys): 31 g/day (25)
Calcium (mg/day)	603.7±162.40	−196.30	57.60	−57.60	4–8 years (both sexes): 800 mg/day (26)
	616.0±94.09	−484.00	29.70	−29.70	9–13 years (both sexes): 1,100 mg/day (26)
Iron (mg/day)	8.4±1.88	4.26	0.67	−0.67	4–8 years (both sexes): 4.1 mg/day (27)
	8.5±0.82	2.79	0.09	−0.51	9–13 years (boys): 5.9 mg/day (27)
	8.9±0.70	3.02	0.72	−0.03	9–13 years (girls): 5.7 mg/day (27)
Zinc (mg/day)	6.1±1.21	2.05	0.43	−0.43	4–8 years (both sexes): 4 mg/day (27)
	6.0±0.51	−0.98	0.16	−0.16	9–13 years (both sexes): 7 mg/day (27)

The values presented as the means±SD. CI=confidence interval, Mean diff=mean difference, ND=not determinable, SD=standard deviation.

In addition, the correlations between zinc intake and energy and macronutrients intakes were all positive for the population studied (Table 2). However, serum zinc showed no correlation with energy and macronutrients, exception to protein intake, in this population (Table 2).

**Table 2:** Correlations among (A) zinc intake versus energy, protein, fat, carbohydrate, fiber, calcium, and iron intakes, (B) serum zinc versus energy, protein, fat, carbohydrate, fiber, calcium, iron, and zinc intakes, and (C) serum zinc versus weight-for-age, height-for-age, BMI-for-age

A	Zinc intake versus							
	
	Energy intake	Protein intake	Fat intake	Carbohydrate intake	Fiber intake	Calcium intake	Iron intake	

*r*	0.3199	0.2878	0.5026	0.2257	0.1462	0.3501	0.3713	
*p*	0.0019*	0.0054*	0.0001*	0.0305*	0.0465*	0.0006*	0.0003*	

B	Serum zinc versus							
	
	Energy intake	Protein intake	Fat intake	Carbohydrate intake	Fiber intake	Calcium intake	Iron intake	Zinc intake

*r*	0.1204	0.2543	0.0211	0.0009	0.0000	−0.0475	−0.0645	−0.0714
*p*	0.2530	0.0145*	0.8417	0.9255	0.9998	0.6528	0.5407	0.4988

C	Serum zinc versus							
	
	Weight-for-age			Height-for-age			BMI-for-age	

*r*	0.0110			−0.0255			0.0027	
*p*	0.9003			0.7722			0.9750	

Statistical parameters after correlation analysis: *r*=Spearman *r*

* *p* = significant.

### Functional indicators

There were 59 boys (mean age 8.3±0.9 years) and 72 girls (mean age 8.4±0.8 years) in the range of 6–9 years of age. The children had weights (25.61±4.47 kg), heights (127.80±7.23 cm), and BMIs (15.54±1.52 kg/m^2^) within the normal reference range for age and gender. Therefore, all children were eutrophic according to BMI and age classification. There was no correlation between anthropometric categories (weight-for-age, height-for-age, and BMI-for-age) and serum zinc (Table 2).

### Biochemical indicators

#### Zinc

**Fig. 2:**
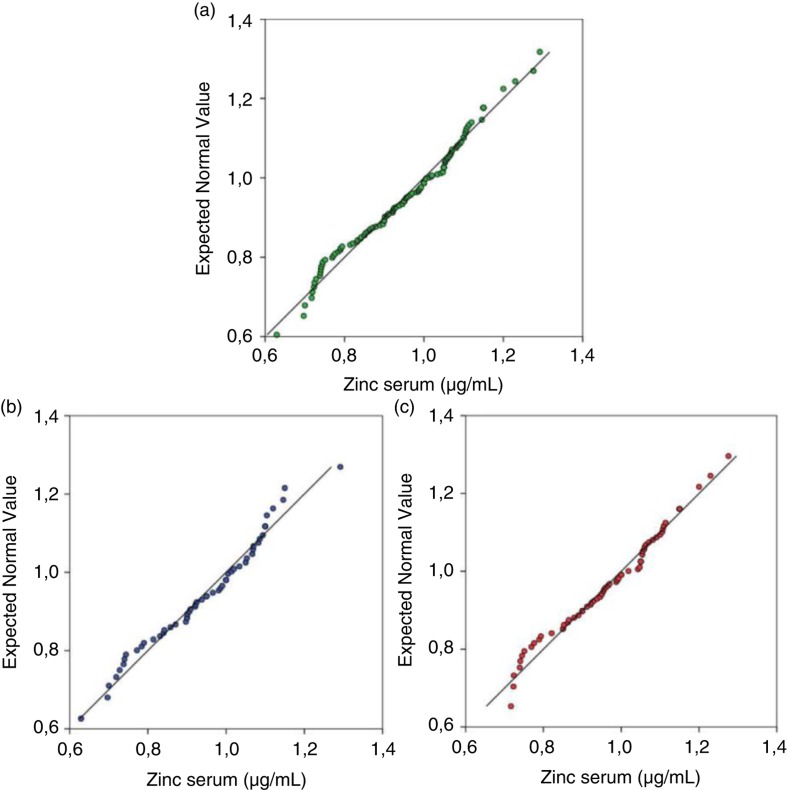
Distribution of basal serum zinc concentrations on children using normal Q-Q plot. (a) In total population (*n*=131). (b) In boys (*n*=59). (c) In girls (*n*=72).

Basal serum zinc concentrations showed normal Gaussian distribution for both genders (Fig. 2). Overall, there was no difference between the percentiles of boys and girls, and the values of 5th and 95th percentiles were 0.72 and 1.15 µg/mL, respectively, for the total population (Table 3). Moreover, the children studied showed no clinical signs of zinc deficiency.

**Table 3:** Reference intervals for basal serum zinc concentration (µg/mL)

		Percentile						
						
	*n*	5th	50th	95th	Minimum	Maximum	Mean±SD	*P-*value
Boys	59	0.70	0.97	1.14	0.63	1.29	0.95±0.14	
Girls	72	0.73	0.98	1.17	0.72	1.28	0.97±0.13	*p*=0.3032
Total population	131	0.72	0.97	1.15	0.63	1.29	0.96±0.13	

*n*=number of children, *p*=not significant, comparing boys and girls.

### Total proteins and fractions

Total proteins (6.66±0.70 g/dL), albumin (3.94±0.57 g/dL), globulin (2.72±0.66 g/dL), and albumin/globulin ratio (1.66±1.04) were within normal reference values. These different proteins were analyzed by the multivariate stepwise regression model and it showed that albumin, globulin, and albumin/globulin ratio correlated positively with serum zinc (Table 4). In addition, 11% of serum zinc variance was justified by the serum albumin concentration, indicating that for each 1 µg/mL of zinc, albumin concentration increased 0.075 g/dL.

**Table 4:** Correlations among serum zinc versus total proteins, albumin, globulin, and albumin/globulin ratio

	Serum zinc versus			
	
	Total proteins	Albumin	Globulin	Albumin/globulin ratio
*r*	0.0951	0.3352	0.3906	0.3016
*p*	0.4981	0.0141*	0.0041*	0.0043*

Statistical parameters after univariate linear regression model: *r*=0.4, α (bilateral)=0.05, β=0.10, and *p=significant.

## Discussion

This study provides reference interval for serum zinc concentration in total population of 0.72–1.15 µg/mL, according to the criteria recommended by the Clinical and Laboratory Standards Institute document C28-A3 (28). These results provide important information about the status of zinc in a sample of a healthy population, considering dietary, functional, and biochemical indicators, that has not been well-described in the literature to date (12, 29, 30).

### Dietary indicators

Serum zinc is the most useful biomarker to assess zinc status in populations (12, 13). Serum zinc concentration reflects recent or habitual zinc intake (14, 31, 32), which means that populations with a diet low in zinc may have lower zinc content in their serum, indicating risk of zinc deficiency (1, 6). Therefore, a quantification of food intake was performed in our children because the assessment of zinc intake and serum zinc should always be performed concurrently (1, 3, 12). Our results showed that energy, protein, fat, carbohydrate, iron, and zinc intakes were all within the expected ranges for age and sex of the children studied. The zinc intake was not different between boys and girls, and there was no positive or negative correlation between serum zinc and zinc intake. In this sense, these findings corroborated the reports of other authors (11, 29). However, there was a positive correlation between serum zinc and protein intake and serum albumin, globulin, and albumin/globulin ratio. Also, there was a positive correlation between zinc intake and energy and macronutrient intakes (protein, fat, carbohydrate, fiber, calcium, and iron) in the population studied. Additionally, fiber and calcium intakes were below the expected ranges for age and sex, which associated to protein sources of animal origin could have enhanced the absorption of zinc in the children studied (6).

Our results are relevant because the prevalence of inadequate zinc intakes seems high in the global population and of great public health concern (1). Moreover, serum zinc reflects the habitual intake of this micronutrient in the diet, and it is influenced by other micronutrients (calcium, iron, and copper), energy, and macronutrients (protein, fat, carbohydrate, and fiber) (14, 33, 34).

### Functional indicators

It is also important to assess anthropometric parameters, in addition to food intake and biochemical parameters, in the evaluation of zinc status (14). Thus, weight-for-age, height-for-age, and BMI-for-age were assessed and were found to be within the normal reference ranges, showing the healthy and homogeneous character of the children studied. Moreover, there was no correlation among these functional indicators with serum zinc. In this sense, these functional indicators may be associated with zinc status, but they are not specific to zinc status and limits in quantifying the prevalence of zinc deficiency (3). For instance, height-for-age is a more appropriate indicator because longitudinal growth is associated with increased zinc intake, whereas the weight-for-age is more associated with the longitudinal growth (3). Moreover, for population assessment, the prevalence of height-for-age must be at least 20% to suggest risk of zinc deficiency (3).

### Biochemical indicators

#### Zinc

Several studies have been published suggesting reference interval for serum or plasma zinc. These studies have had a wide age range from <1 year to >70 years of age, without sample homogeneity, as summarized in Table 5.

**Table 5:** Values of zinc concentration expressed in different ways and in different age groups

Age	Gender	Cutoff (µg/mL)	Mean±SD (µg/mL)	Reference range (µg/mL)	Total population (µg/mL)	Reference
Birth–13 years	Male and Female			0.42–1.40		Brown et al. (15)
3–9 years	Male and Female		0.83±0.003			Hotz et al. (4)
3–18 years	Male		1.14±0.35		1.14±0.36	Ghasemi et al. (29)
	Female		1.12±0.37			
7–10 years	Male		0.88±0.15 (7–8 years)			Lin et al. (14)
	Female		0.93±0.16 (9–10 years)			
>10 years	Male	0.74				Hess et al. (12)
	Female	0.70				

Our results showed different reference intervals from these reports in the literature. The boys had interval of 0.70–1.14 µg/mL and girls had interval of 0.73–1.17 µg/mL. The interval in the total population was 0.72–1.15 µg/mL, with a mean value of 0.96±0.13 µg/mL. In this sense, we observed how the serum zinc concentrations changed between different publications, probably because of confounders or geographic regions.

Regarding the gender of the children, the literature shows conflicting basal values of serum zinc. Some authors have reported higher values for boys than girls in the prepubertal and pubertal stage (4, 35), while other authors have shown no difference (6, 14). Our results showed no difference between genders, similar to Lin et al. (14), emphasizing that our results have a Gaussian distribution.

The values of the reference intervals obtained in our study are important from a nutritional point of view because they were obtained in a homogeneous sample of children from a region of northeastern Brazil (14). We emphasize the regional study because Hambidge et al. (36), for the first time, reported the existence of zinc deficiency in children in a population of 338 apparently normal subjects living in Denver (USA) with ages ranging from 0 to 40 years. In addition, this study provides more information related to young healthy children, which is important because reports in the literature are insufficient to draw firm conclusions about the global prevalence of zinc deficiency in children (6, 13). It should be emphasized that serum zinc is a good biomarker for population zinc status (6). Therefore, these values of the reference intervals are important for the interpretation of the risk of zinc deficiency and its clinical management. And when the prevalence of low serum zinc concentration is higher than 20%, we have a serious public health problem (31, 37). In this regard, additional studies to establish reference intervals are needed, particularly in children and adolescents for whom there are limited data (29, 30).

### Total proteins and fractions

Serum concentrations of total proteins are also an important biochemical parameter to assist in the investigation of zinc status, because their serum changes can promote changes in serum zinc, as 70% of this nutrient is transported in the blood primarily bound to albumin, α-2-macroglobulin, transferrin and ceruloplasmin (6). However, total serum proteins showed no changes in our children. The fact that there was a positive correlation between protein intake and serum zinc means that efficient zinc absorption is dependent on dietary protein intake and digestion (38). Moreover, there was a positive correlation with serum zinc and serum albumin and globulin, confirming the close relationship between these ions and its main blood carrier (5).

### Limitations

The main limitations of our study were the impossibility of increasing the sample number of children because of logistic, 24-h dietary recall, and the refusal of the children to participate in the study. In addition, there was no measurement of acute inflammatory factors like C-reactive protein and α-2 macroglobulin.

## Conclusions

The total population studied showed the reference interval of 0.72–1.15 µg/mL for a serum zinc concentration that can be used to establish the zinc status in a specific population. Although the serum zinc has positively correlated only with the protein intake and serum protein fractions, in healthy children, we consider it important to use three indicators (dietary, functional, and biochemical) to establish the zinc status (3).
